# Lung Cancer Disparities in Hispanics: Molecular Diagnosis and Use of Immunotherapy

**DOI:** 10.1200/GO.20.00004

**Published:** 2020-06-08

**Authors:** Luis E. Raez, Andrés F. Cardona, Oscar Arrieta, Gilberto Lopes

**Affiliations:** ^1^ Hematology/Oncology Department, Memorial Cancer Institute/Florida International University, Miami, FL; ^2^ Clinical and Translational Oncology Group, Lung Cancer Unit, Clínica del Country; Foundation for Clinical and Applied Cancer Research; and Molecular Oncology and Biology Systems Group, Universidad El Bosque, Bogotá, Colombia; ^3^ Thoracic Oncology Unit and Laboratory of Personalized Medicine, Instituto Nacional de Cancerología, Mexico City, Mexico; ^4^ Global Oncology Department, University of Miami Sylvester Comprehensive Cancer Center, Miami, FL

There are > 50 million people in the United States who identify themselves as Hispanic, and we have > 20 countries with Hispanic population in Latin America (LATAM). Lung cancer is a public health problem worldwide and Hispanics are not exempt. There are disparities in the outcomes of Hispanics compared with non-Hispanic Whites (NHWs), such as better survival despite inferior access to care or higher rate of *EGFR* mutations. In addition, there is underrepresentation of Hispanics in lung cancer studies, resulting in a need to research and validate the findings seen in NHWs. Another particular challenge for Hispanics is the fact that they are not a race but a diverse group of people with regional and cultural differences that can contribute to disparities. In addition, the lack of adequate resources to fight lung cancer creates more disparities in the diagnosis and treatment of patients with lung cancer in LATAM. Worldwide, lung cancer is the most common malignancy and the most frequent cause of cancer deaths.^1^ In 2019, approximately 2.1 million new diagnoses were made, accounting for 11.6% of the total cancer incidence burden.^2^ According to the Global Burden of Disease study 2020,^3^ the health care burden and costs attributed to lung cancer were substantial on a global scale. The 5-year survival rate of lung cancer (17.8%) was much lower than that of other leading cancers.^2^ As a result of the high fatality rate (83%),^4^ its geographical mortality patterns closely follow those of incidence, and it remains to be an important public health issue. Lung cancers kill more people in Latin America (LATAM) than any other malignancy. According to the International Agency for Research on Cancer, in 2012, just more than 60,000 people died of lung cancer in the LATAM region. This represents > 10,000 more lives lost than the next most lethal cancer and approximately 11%-12% of all neoplasm deaths.^3^

Variation in cancer patterns, such as incidence and mortality, between similar populations across different geographic locations arises from changes in risk as a result of the interaction between genetics and environmental exposures to carcinogens. Cancer mortality, although largely influenced by this underlying risk, is also a function of survival among those who develop cancer. Migrant studies, comparing cancer outcomes between similar generations of immigrant populations and their respective country of origin, may shed light on the gene-environment interaction for different cancers and provide insights into differences in access to and quality of treatment.^5^ According to the US Census Bureau, the Hispanic/Latino population in the United States is estimated to be 18.3% (58.9 million in 2018), and it is projected to make up 31% of the population by 2060. However, the Hispanic population is composed of a diverse group of individuals who trace their heritage to > 20 Spanish-speaking countries, regardless of race.^6^ When compared with the non-Hispanic White (NHW) population, Hispanics tend to experience greater health disparities as a result of structural, sociodemographic, psychosocial, and cultural factors.^7^ Indeed, approximately 24% of Hispanics live below the poverty line,^8^ and 35% have less than a high school education.^9^ In addition, in 2012, one third of US Hispanics had no health insurance and reported not having a consistent health care provider.^10^ The incidence of lung cancer in LATAM depends of the country; it can be as low as 5.9 per 100,000 people in Bolivia to as high as 29 per 100,000 people in Uruguay. In the United States, according the American Cancer Society (ACS), the incidence is 39.2 per 100,000 for Hispanic males and 24.6 per 100,000 for Hispanic females.^11^ Although Hispanics have an overall lower incidence for all cancers pooled and there were reports from the American Lung Association, ACS,^12^ and others that they have a better survival compared with other minorities (Hispanic paradox), recently, Pinheiro et al^5^ reported that mortality from tobacco-related cancers among migrants is unsurprisingly higher among one Hispanic group—Mexican Americans—given their higher smoking prevalence.^13^ In women, lung cancer mortality is substantially higher in both Mexican immigrants and Mexican Americans.^14,15^

Usually, most lung cancer studies are done in NHW populations in the United States or Europe; however, in the past few years, the field of thoracic oncology research has observed a large number of studies coming from Asia (China, Korea, and Japan). So far, few studies have explored the behavior of lung cancer among Hispanics in the United States or LATAM, studying whether it is the same as in other populations. For now, we know that LATAM has a history of extensive mixing between Native Americans and people arriving from Europe and Africa.^16^ As a result, individuals in the region have a highly heterogeneous genetic background and show great variation in physical appearance and disease incidence and prevalence. In addition, Hispanics have a better lung cancer survival than other minorities, perhaps because of the genotypic heterogeneity of its origin.^17^

Current evidence has strongly suggested that ethnicity might be a risk factor for *EGFR-*mutant lung cancer, but there is no direct evidence from an admixed population. Analyses of *EGFR* mutation frequencies have showed varying rates in LATAM countries (approximately 15% in Argentina; 20%-25% in Brazil; 25%-35% in Mexico, Costa Rica, and Colombia; and 55% in Peru).^18-20^ Interestingly, the population in Peru is mostly of Native American descent (with some influence of the migrations of East Asia; ie, China and Japan), whereas the Brazilian, Mexican, Costa Rican, and Colombian populations are mixed.^18^ These observations suggest that somatic mutation frequency in *EGFR* in lung cancer could be associated with genetic ancestry. In contrast, Argentina and Uruguay have the lowest rates of *EGFR* mutations in LATAM, a finding related to a strong history of Italian and European immigration and perhaps the higher tobacco consumption rates in these countries.^21^ According to the GLOBOCAN database, lung cancers in women range from 20% of the total patients in Paraguay to as high as 50% of the patients in Peru and Bolivia, whereas the incidence of women with lung cancers with *EGFR* mutations in LATAM can range from 25% to 60% in Peru.^18^ In most LATAM countries, we see more *EGFR* mutations in women compared with men. Another regional study explored the molecular epidemiology of *ALK* translocations, finding that their incidence is as low as in the NHW population of the United States and Europe.^22^

However, LATAM countries still have important access barriers for comprehensive molecular testing (next-generation sequencing [NGS]) in non–small-cell lung cancer (NSCLC). A recent evaluation of 4,389 patients from various countries around the world showed that oncologists in the United States, Europe, Japan, and LATAM request one or more molecular tests in lung cancer in 97%, 79%, 90%, and 76% of patients, respectively.^23^ However, in LATAM, it is uncommon to see testing for an actionable gene other than *EGFR* or *ALK*. *EGFR* (the only biomarker available for targeted therapy 10 years ago) is still one of the most frequently tested biomarkers across countries, but it is more frequently tested in the United States than elsewhere, and in every region, it is now less frequently tested than PD-L1, probably because it is easier to perform immunohistochemistry than NGS or polymerase chain reaction. This has profound implications on the way oncologists decide to treat their patients because PD-L1 and EGFR are not exclusive.

The next step was to evaluate what happens when this Hispanic population migrates to the United States. Raez et al^24^ performed a genomic analysis of 492 patients with NSCLC who live in Florida, finding that Hispanics living in the United States seem to have a higher rate of *EGFR* mutations (approximately 25%) compared with NHWs. However, when they matched this Hispanic population to the NHWs who live in South Florida, there were no statistically significant associations except more patients with *EGFR* exon 19 mutations among Hispanics than NHWs. Likewise, if we consider the main outcomes to immunotherapy in NSCLC, most of the CheckMate and KEYNOTE trials were done in the United States and Europe, and they did not include or included a minimal number of Hispanics. Recently, Raez et al^25^ reported information from 256 Hispanic patients with NSCLC treated with immunotherapy (mainly as second-line therapy) in Argentina, Peru, and the United States (Miami). In parallel, the authors matched these data with 180 NHW controls, finding no difference in outcomes (progression-free survival [PFS] and overall survival [OS]) at 4 and 22 months. Nevertheless, this study also reported a lower overall response rate in Hispanics versus NHWs in patients receiving second-line therapy (18% *v* 19%), patients with adenocarcinomas (22% *v* 24%), and patients with PD-L1–positive tumors (29% *v* 32%); however, none of these differences were statistically significant. Another study, the Quijote study,^26^ include 296 Hispanic patients (from México, Costa Rica, Nicaragua, Panama, Colombia, Peru, the United States, and Argentina) with NSCLC treated with immunotherapy in the first-, second-, or third-line or greater setting (13.7%, 48.8%, and 37.6% of patients, respectively). Within this population, the median OS was 19.9 months (95% CI, 14.5 to 22.7 months), and median PFS was 3.73 months (95% CI, 2.8 to 4.2 months). In addition, the factors associated with increased survival included treatment in the first-line setting (*P* < .001), complete or partial response (*P* < .001), and positive PD-L1 status (*P* = .0039). Compared with a historical cohort, immunotherapy proved to be superior in terms of OS (*P* = .05) but not PFS (*P* = .2). The Quijote study^26^ also documented 44 patients with hyperprogressive disease (19.8%; 95% CI, 14.5% to 25.1%) who had a median time to progression after immunotherapy of approximately 5 weeks. Factors associated with worst outcomes among Hispanic patients with hyperprogressive disease included albumin and hemoglobin levels, presence of CNS and bone metastasis, and weight loss. The study also revealed that a leukocyte count > 5,300 cells/dL was present in all patients with hyperprogressive disease.^27^

Lung cancer disparities in Hispanics are also related to other factors such as restricted open access to targeted therapy. Lenz et al^28^ evaluated 1,735 patients with *EGFR* mutation–positive metastatic lung adenocarcinoma in Brazil. The authors estimated that, if treated with chemotherapy, only 71 of patients would be free of progression after 24 months. In contrast, if all of the patients were treated with anti-EGFR tyrosine kinase inhibitors, the expectation was that 24-month PFS would be achieved in 312 patients treated with erlotinib, 377 patients treated with gefitinib, and 388 patients treated with afatinib. However, the reality in Brazil is that only a minority of the population (< 25%) has access to more complex treatments, especially in the area of oncology. Similarly, Goncalves et al^29^ established that 2,332 premature deaths would occur in Brazilian patients with advanced NSCLC 1 year after diagnosis, exclusively because of the lack of access to immunotherapy. Dramatically, when comparing Brazilian incidence data with SEER data, these numbers can reach 11,193 premature deaths in a single year.

An important issue is that the Hispanic population is generally not represented in lung cancer clinical trials because of limited access and few research centers. It is likely that there are differences regarding survival and quality of life of patients treated in the community oncology center compared with those treated in the scope of clinical trials. Barrios et al^30^ evaluated the difference in survival of patients with stage IV lung cancer, comparing patients treated in the public health system of a Brazilian university cancer center versus a research center of the same institution. Forty-one patients were treated in the public health system and 66 patients in the research center, and the study demonstrated an impressive difference in OS. The median OS times were 5 and 10 months in patients treated in the public health system versus the research center, respectively (hazard ratio [HR], 0.6; 95% CI, 0.37 to 0.95; *P* = .03). These data were confirmed in Mexico, where the enrollment in any lung cancer clinical trial was associated with a better OS (HR, 0.47) independent of other prognostic factors.^31^

In conclusion, important disparities in lung cancer care exist, not only between developing countries and high-growth economic nations, but also between poor and rich inhabitants of developing countries.^32^ There are evident differences between a recognizable standard of cancer care and the treatment available in the diverse health systems for the populations of LATAM. It is possible to point out differences from the recommendations of international guidelines in every step of cancer care, from the diagnosis to the treatment of NSCLC in advanced disease. Because of the differences in treatment between guidelines and public services, 25% of the inhabitants of the largest country in LATAM (Brazil) pay for private health insurance. Despite the existence of several biomarkers for lung cancer, limited reimbursement exists in LATAM for their use. *EGFR* mutation and *ALK* translocation testing is done in private laboratories, and usually, pharmaceutical companies sponsor the test.^33^ Across LATAM countries, it is estimated that < 30% of patients with lung adenocarcinomas are tested for *EGFR* and only approximately 10% are tested for *ALK* fusion. Some reasons for the low rates of testing are the difficulty in access and the cost of the test, small tissue and quality of biopsy samples, and issues regarding limited access to the drugs needed in these scenarios. It is time to change this regional situation. It is certainly in our hands, but because Hispanics are a heterogeneous group, not only in LATAM but also in the United States, we will have to enact a diverse group of approaches to improve the outcomes of our Hispanic patients with lung cancer.
